# Positive end-expiratory pressure improves oxygenation inducing ventral-to-dorsal tidal ventilation redistribution: an electrical impedance tomography study

**DOI:** 10.1186/cc9610

**Published:** 2011-03-11

**Authors:** T Mauri, G Bellani, A Pradella, A Grassi, P Tagliabue, M Bombino, N Patroniti, G Foti, A Pesenti

**Affiliations:** 1 Universita degli Studi di Milano-Bicocca, Monza, Italy; 2 San Gerardo Hospital, Monza, Italy

## Introduction

Positive end-expiratory pressure (PEEP) improves oxygenation in acute lung injury (ALI) patients by increasing end-expiratory lung volume (EELV). Electrical impedance tomography (EIT) is a relatively new non-invasive bedside method to monitor regional distribution of tidal ventilation and EELV changes, validated in preclinical studies. We tested EIT as a monitor of PEEP-induced tidal redistribution and EELV changes in ALI patients, and the relationship between EIT parameters and oxygenation.

## Methods

We enrolled 14 consecutive ALI patients admitted to our ICU, intubated and undergoing mechanical ventilation. We monitored the regional tidal ventilation distribution by means of a new EIT system (PulmoVista 500^®; ^Dräger Medical GmbH, Lübeck, Germany) dividing the lung imaging field into four contiguous same-size regions of interest (ROIs): ventral right (ROI 1) and left (ROI 2) and dorsal right (ROI 3) and left (ROI 4). EIT allowed us to measure changes in EELV at different PEEP levels by measuring differences in end-expiratory total lung electrical impedance. We randomly performed the following two steps for 15 minutes, leaving tidal volume and FiO_2 _unchanged: PEEP_low _(clinical) and PEEP_high _(PEEP_low _+5 cmH_2_O). At the end of each step, we recorded: ventilation parameters, arterial blood gas analysis, percentage of tidal ventilation distribution in the four ROIs and EELV change. Analyses were performed by paired *t *test and linear regression.

## Results

Patients were 55 ± 12 years old and seven were women. ALI etiology was: trauma (14%), septic shock (21%), pneumonia (37%) and postoperative respiratory failure (28%). PEEP_low _was 7 ± 2 cmH_2_O and PEEP_high _12 ± 3 cmH_2_O. At PEEP_high_, PaO_2_/FiO_2 _significantly ameliorated (266 ± 98 vs. 287 ± 102 mmHg, *P *= 0.0003), the proportional distribution of tidal ventilation changed in all four ROIs (ROI 1 to ROI 4: 34 ± 14 vs. 29 ± 9%, *P *= 0.03; 33 ± 13 vs. 30 ± 11%, *P *= 0.12; 16 ± 9 vs. 20 ± 10%, *P *= 0.002; 17 ± 7 vs. 21 ± 6%, *P *= 0.002), moving from ventral to dorsal, and EELV increased by 349 ± 121 ml. Changes in PaO_2_/FiO_2 _correlated better with ventral-to-dorsal shifts of tidal ventilation than with EELV changes (*r *= 0.499, *P *= 0.08; *r *= -0.399, *P *= 0.18).

## Conclusions

EIT allowed us to detect ventral-to-dorsal tidal ventilation redistribution at higher PEEP levels. This mechanism may be a key determinant of PEEP-induced oxygenation improvement.

